# Innovation Experiences from Africa-Led Drug Discovery at the Holistic Drug Discovery and Development (H3D) Centre

**DOI:** 10.1021/acsmedchemlett.2c00142

**Published:** 2022-07-11

**Authors:** Vinayak Singh, Dickson Mambwe, Constance Mawunyo Korkor, Kelly Chibale

**Affiliations:** # Drug Discovery and Development Centre (H3D), University of Cape Town, Rondebosch 7701, South Africa; † Department of Chemistry, University of Cape Town, Rondebosch 7701, South Africa; § South African Medical Research Council Drug Discovery and Development Research Unit, Department of Chemistry and Institute of Infectious Disease and Molecular Medicine, University of Cape Town, Rondebosch 7701, South Africa

**Keywords:** Malaria, tuberculosis, drug discovery and development, chemical matter, clinical trial

## Abstract

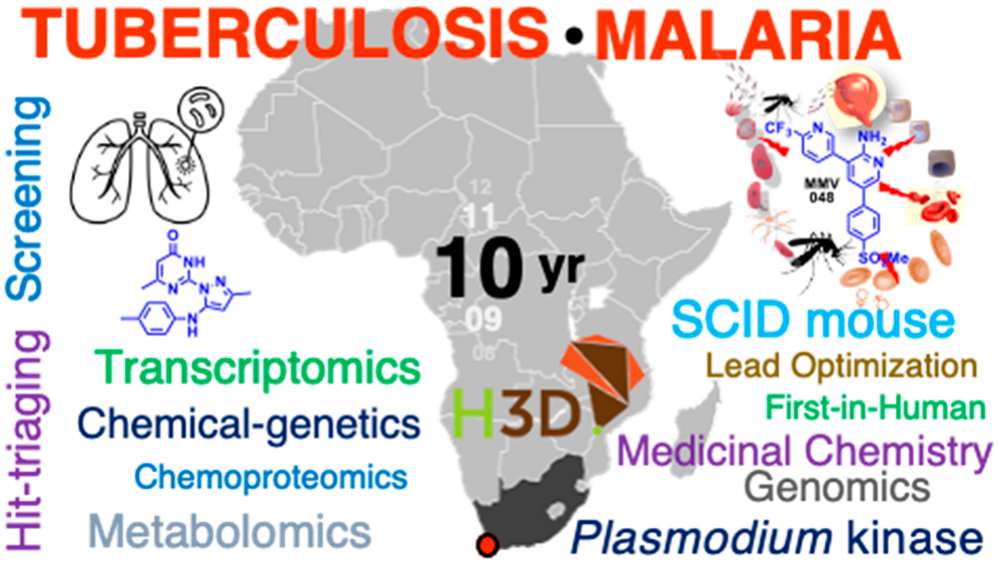

As the so-called “next frontier” in global economic terms, Africa’s disease burden continues to choke and cripple economic growth across the continent. The highest burden is attributable to malaria and tuberculosis (TB), which also remain among the deadliest infectious diseases affecting mankind the world over (Malaria, 627,000 deaths; TB, 1.5 million deaths, in 2020). In achieving self-determination with respect to the health needs of all who live on the continent, Africa must align with global north efforts and be a source of health innovation. This will in part require the creation of an ecosystem of innovative pharmaceutical R&D and expanding it across the continent by scaling up through sustained performance and excellence. To this end, the Holistic Drug Discovery and Development (H3D) Centre at University of Cape Town in South Africa has risen to this challenge. Here, we highlight the innovation experiences gained at H3D, covering the advances made in our quest to contribute to a global pipeline of therapeutic interventions against malaria and TB. We discuss selected chemical series starting from their identification, structure–activity relationships, mode of action, safety, proof-of-concept studies, and lessons learned.

Africa represents 15% of the global population and bears 25% of the global disease burden.^1^ Such diseases include malaria and tuberculosis (TB), both infectious diseases of high mortality and morbidity on the continent. According to the World Health Organization (WHO) *World Malaria Report* 2021, using new analysis methodology, there were 627,000 malaria deaths in 2020, with 14 million more people contracting malaria and 69,000 more dying from it than the year before.^2^ These statistics in part reflect the contribution from the disruption of malaria services as a result of the COVID-19 pandemic. However, even without factoring in the pandemic, the new methodology reveals that there were some 558,000 malaria deaths globally in 2019, nearly 150,000 deaths more than previous estimates. Currently, antimalarial chemotherapy using frontline artemisinin combination therapy regimens forms the cornerstone for the treatment of malaria. Although the use of this combination therapy has resulted in a significant decrease in the global malaria incidence, reports of reduced sensitivity of *Plasmodium falciparum* (*Pf*) to artemisinin derivatives poses a potential threat to their continued efficacy and to malaria control and subsequent eradication.^3^

Similarly, the insidious TB scourge is only second to the recent COVID-19 pandemic in claiming lives compared to other infectious diseases. A record high TB mortality rate stood at an estimated 1.3 million in 2020 and continues to be further worsened by co-infection with the human immunodeficiency virus among other comorbidities, especially in the endemic African regions. The progress made in TB control over the past decade, and possibly beyond, has been reversed and is threatened by the shift in resources toward COVID-19, as recently reported by the WHO.^4^ The cornerstone of treatment and control of drug-sensitive TB has for a long time been underpinned by the so-called “short-course” combination therapy with a 6 month treatment duration. This treatment regimen consists of an initial intensive phase in which a combination of isoniazid, ethambutol, pyrazinamide, and rifampicin is taken for 2 months to achieve significant bacterial load suppression. To prevent bacterial recrudescence, this phase is rapidly followed by a continuous phase of isoniazid and rifampicin for 4 months. The long treatment duration reduces patient adherence, diminishes drug efficacy, and significantly contributes to the emergence of drug resistance.^5^

The development of novel drugs such as bedaquiline and repurposing of clofazimine for TB^6^ provide for shorter treatment regimens and hope for the management of the disease burden. However, these are not without limitations, not to mention the inevitable and ever-present threat of drug resistance.^7^ Currently, global efforts are aimed at delivering novel antimalarial and anti-TB drugs that are devoid of the liabilities associated with the current regimens. While the complex life cycle of human malaria parasites and development of drug resistance are the main obstacles in malaria control and potential eradication, TB presents its own unique challenges including dormant cells, long duration of treatment, and emergence of multidrug resistance. Although in the case of TB there is a BCG vaccine available which provides limited protection to children, a malaria vaccine for use in the general population is yet to be developed. However, some progress has been made with the development of the RTS,S/AS01 malaria vaccine, which was recently recommended by the WHO for use in children from 5 months of age living in regions with moderate to high transmission.^8^ It is noteworthy that this vaccine is only about 40% effective.

The urgency to deliver new drugs for both malaria and TB has over the years prompted the formation of various innovative product development partnerships (PDPs), such as the Medicines for Malaria Venture (MMV),^9^ the TB Alliance,^10^ as well as precompetitive drug discovery consortia exemplified by the Malaria Drug Accelerator (MalDA)^11^ and the TB Drug Accelerator (TBDA).^12^

In the context of Africa-led drug discovery, the Holistic Drug Discovery and Development (H3D) Centre based at the University of Cape Town (UCT) in South Africa is a key African partner in the MalDA and TBDA consortia. H3D was founded in 2010 as a UCT-accredited research center and was officially launched in April of 2011. As the first and only one of its kind on the African continent, H3D is an integrated drug discovery platform whose vision is to be a leading organization for drug discovery and development. The mission of H3D is to discover and develop innovative life-saving medicines for diseases that predominantly affect African patients. H3D is also focused on building Africa-specific models aimed at improving treatment outcomes in African patients and on education and training of a critical mass of skilled African-based drug discovery scientists. This article will showcase the progress we have made in malaria and TB drug discovery through collaborations with a global network of partners from industry, academia, PDPs, philanthropic organizations, and the South African government. At this juncture, it is noteworthy that, in these collaborations, the projects were conducted in Africa and led by H3D. It was important and advantageous to have the projects conducted in Africa for three main reasons. First, due to the high burden of malaria and TB in Africa with attendant consequences both on the health and socioeconomic development of the continent, it is important for African-based scientists to take a leading role in drug discovery against these diseases. Second, there is a strong interplay between genetics, the socioeconomic and physical environment in which patients live, and effective treatment of disease. For this reason, it is vital to conduct drug discovery and development campaigns in close proximity to African patient populations to understand and meet the pressing health needs brought about by malaria and TB. Third, conducting the project in Africa was important to build drug discovery capacity as a secondary objective so as to engage with the capability developed sustainably in the longer term. On the other hand, the initial disadvantages of the project being done in Africa revolved around limited access to drug discovery infrastructure, technology platforms, experience, and a limited pool of appropriately skilled scientists exacerbated by the continued brain drain. Some aspects of our work can also be found in our recent publication: “*Medicinal Chemistry out of Africa*”.^13^

## Malaria

**Figure 1:**
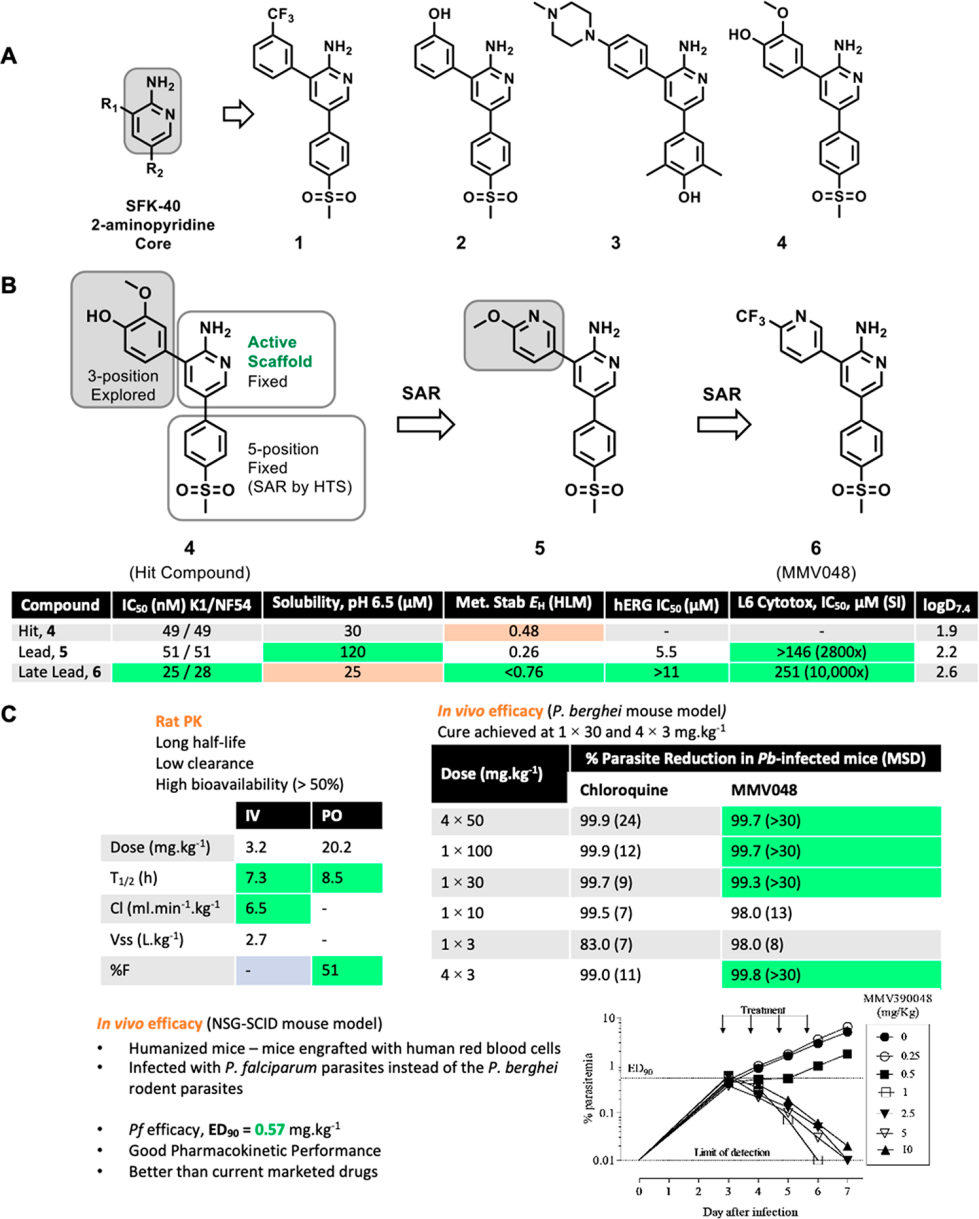
Evolution of MMV048. (A) Hit identification and validation of representative SFK-40 3,5-diaryl-2-aminipyridine hits. (B) Hit to lead and lead optimization. (C) *In vivo* rat PK and mouse efficacy studies (*P. berghei* and humanized NOD-*scid IL-2R*_*γ*_^*null*^ mice). K1, multidrug resistant strain of *P. falciparum*; NF54, drug susceptible strain of *P. falciparum*; HLM, human liver microsomes; L6, cell lines derived from rat skeletal muscle; MSD, mean (mouse) survival in days.

Phenotypic whole-cell high throughput screening of a 36,608-member SoftFocus^14^ Kinase (SFK) library of small molecules, spanning more than 200 chemotypes, against the human malaria parasite *Pf* drug-sensitive (3D7) and drug-resistant (Dd2) strains led to the identification of more than 200 hits displaying selective antiplasmodium activity. A greater than 80% inhibition of parasite growth at a primary and retest concentration of 1.82 μM and absence of cytotoxicity on a mammalian cell-line at this concentration was defined as hit criteria. Within this library, chemotypes which delivered hits included the SFK-40 sublibrary of 3,5-diaryl-2-aminopyridines exemplified by compounds **1**–**4** (Figure 1). Compound **4** was consequently selected and prioritized for further cell-based medicinal chemistry optimization following in-house hit resynthesis, retesting against *Pf Pf*NF54-drug sensitive (NF54) and *Pf*K1-multidrug resistant (K1) strains *in vitro*, and absorption, distribution, metabolism, and excretion (ADME) profiling.

ADME evaluation of compound **4** revealed a metabolic liability evidenced by a high predicted human hepatic extraction ratio (*E*_H_ = 0.48) in human liver microsomes (Figure 1) likely due to the presence of the 2-methoxyphenyl moiety at position 3 of the 2-aminopyridine core. On this basis, a hit-to-lead (H2L) cell-based medicinal chemistry progression of compound **4** was initiated to address the aforementioned liability. This effort led to the identification of compound **5**, an equipotent (IC_50_ ∼ 50 nM) methoxylpyridyl-containing early lead with improved *in vitro* metabolic stability (*E*_H_ = 0.26) and solubility, and demonstrating *in vivo* curative effect at a 4 × 50 mg·kg^–1^ multidose in *Plasmodium berghei* infected mice.^15^

The identification of compound **5** triggered a lead optimization (LO) campaign aimed at exploring potential opportunities and liabilities of this series and optimizing *in vitro* potency, ADME properties, and *in vivo* efficacy to identify a potential clinical candidate for development. This campaign successfully delivered compound **6** (MMV048), a trifluoromethyl analogue of **5** (Figure 1), augmenting the structure–activity relationship (SAR) revealed at the 5-position from the initial HTS screen within the series.^15^

MMV048 had high *in vitro* antiplasmodium potency (IC_50_ = 25 nM), an impressive metabolic stability profile (*E*_H_ < 0.07), and excellent pharmacokinetic (PK) properties (Figure 1). These attributes translated to *in vivo* efficacy in both the rodent *P. berghei* and humanized *Pf*-infected NOD-*scid IL-2R*_*γ*_^*null*^ mouse malaria infection models (ED_90_ = 1.1 and 0.57 mg·kg^–1^, respectively in a four-dose regimen). Compared to early lead (**5**), MMV048 cured *P. berghei*-infected mice at a single dose of as low as 30 mg·kg^–1^ (Figure 1).^15,16^ Both compound **5** and MMV048 exhibited low potential for drug–drug interaction risk, evidenced by their low inhibition (IC_50_ > 20 μM) against all five major cytochrome-P450 (CYP450) isoforms. The two compounds were clean against L6 cells (IC_50_ < 146 μM) with a negative result in the Ames test indicating low risk of genotoxicity. However, the difference was in hERG activity wherein compound **5** showed moderate inhibitory activity (IC_50_ < 5 μM) and MMV048 showed an improved profile (IC_50_ > 11 μM), with no adverse change in electrocardiogram being observed when MMV048 was further evaluated in an *in vitro* rabbit ventricular wedge assay at 2 μM.^15^ Further SAR studies were conducted on both compound **5** and MMV048 to delineate SAR for *in vitro* antiplasmodium and hERG activities. However, the previously observed exceptional curative oral efficacy in the *P. berghei* mouse model could not be reproduced, albeit improvements of *in vitro* antiplasmodium and hERG activities.^16^

MMV048 demonstrated potential for interrupting transmission, with submicromolar potency against gametocytes (IC_50_ < 0.214 μM) in alignment with its observed efficacy against male gametes and oocysts in mosquitoes. Additionally, a moderate reduction in the number of mice that developed blood-stage infection was observed in a host-to-host transmission model with *P. berghei*.^17^ MMV048 displayed potent *in vitro* activity against the *Plasmodium vivax* related simian parasite species, *Plasmodium cynomolgi* (IC_50_ = 0.064 μM), via its prevention of early stage hypnozoite and schizont development in the liver. Evidence of *in vitro*–*in vivo* correlation for its prophylactic properties was demonstrated by its high *in vivo* efficacy in monkeys infected with *P. cynomolgi*.^17^

**Figure 2:**
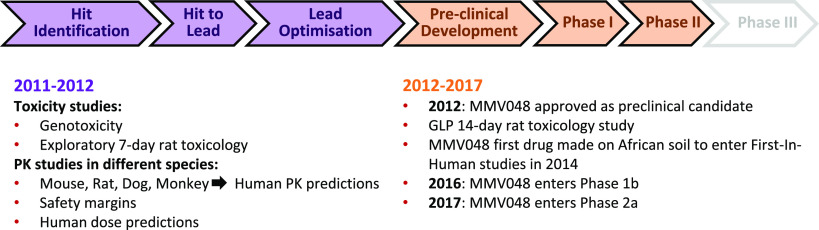
Preclinical development timeline for MMV048.

Following its nomination and approval as a preclinical candidate in 2012 (Figure 2), follow-up studies revealed that MMV048 maintained low clearance, a long half-life ,and good oral bioavailability across rat, dog, and monkey species, thereby validating the observed correlation between the *in vitro* potency and the *in vivo* efficacy. Having been extensively assessed for its toxicity (genotoxicity, GLP 14-day rat and dog exploratory toxicology), MMV048 was approved and progressed to Phase 1 First-in-Human clinical trials where its safety, tolerability, and pharmacokinetic profiles were determined in healthy volunteers in three separate studies. Next, it was investigated in a volunteer infection study using the *Pf* induced blood-stage malaria infection model, in which the compound was well tolerated in humans with pharmacokinetic properties indicating potential for use in chemoprophylaxis.^18^

MMV048’s potential for use as a component of a single-dose combination therapy was shown by its striking 90-h half-life in human PK predictions, with doses as low as 80–100 mg required to maintain a therapeutic concentration over 8-days. Based on this exciting profile, it proceeded to First-in-Human studies.^18^ In these First-in-human studies, the elimination half-life of MMV048 (>149 h) was observed to be longer than predicted in preclinical studies (90 h).

Mechanism of action (MoA) studies involving chemical-genetics and chemoproteomic pull-down studies identified *Pf* phosphatidylinositol-4-kinase (*Pf*PI4K) as the target. It showed impressively high selectivity over human lipid kinases. Inhibition of PI4K was confirmed in a biochemical assay against the *Plasmodium vivax* enzyme (*Pv*PI4K) revealing an IC_50_ of 3.4 nM with a strong correlation between enzyme inhibitory potency and whole-cell antiparasitic activity. PIP4K2C was the only human target protein affected with an IC_50_ value in the same range as that of *Pv*PI4K.^17^ Despite its remarkable progress up to this stage, MMV048 still had a limited solubility liability (Figure 1), which would lead to challenges upstream and further in development requiring reformulation.^15,18^ Additionally, there was room for improvement of its activity against liver- and transmissible gametocyte-stage parasites to potentially achieve a radical cure. Therefore, a campaign was initiated to identify back-up compounds with better physicochemical properties, similar/better pharmacokinetics, and efficacy, as well as differentiated toxicity profile in terms of some human kinase off-targets.

**Figure 3:**
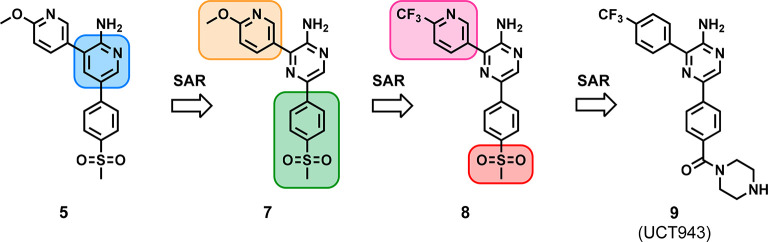
Progressive LO leading to the discovery of compound **9** (UCT943).^19^

Using early lead compound **5** as the starting point, SAR explorations were focused on modifications to the 2-aminopyridine core (Figure 3).^19^ Coupled with learnings from previously established SAR leading to the discovery of MMV048,^15,16^ this campaign delivered compounds **7** and **8**, containing a 2-aminopyrazine core, displaying equipotent activity.^20^ Further rigorous SAR studies based on **8** aimed at improving solubility via introduction of water-solubilizing groups at the 4-position of the 5-phenyl ring (sulfone group replacement) and other strategies led to the identification of compound **9** (UCT943), a piperazine amide derivative with a pyrazine core (Figure 3). The LO assessment package for UCT943 revealed its exceptional single-digit nanomolar *in vitro* antiplasmodium potency (IC_50_ = 5.4 nM), high solubility (158 μM) and a better hERG profile (IC_50_ > 10 μM) while retaining exceptional PK properties as that of MMV048 (Table 1).^19^

**Table 1:** Comparison of Various *In Vitro*, Physicochemical, and *In Vivo* Properties for MMV048 and UCT943

	property	MMV048	UCT943
*In vitro* potency	*Pv*PI4K IC_50_	3.4 nM	23 nM
	*Pf*NF54 IC_50_	28 nM	5.4 nM
	*Pf*K1 IC_50_	25 nM	4.7 nM
physicochemical property	log *D*_7.4_	2.60 (0.01)	–0.27 (0.03)
	thermodynamic solubility (pH)^*a*^	4.2 μg·mL^−1^ (6.5)	110 μg·mL^−1^(6.0)
	Mol wt	393.4 g·mol^–1^	427.4 g·mol^–1^
	p*K*_a_ (measured)	4.0 (0.07)	7.45 (0.05)
cardiotoxicity risk	hERG IC_50_	>11 μM	10 μM
life cycle stage activity	*Pc* liver hypnozoites/schizonts IC_50_	64 nM	<10 nM
	*Pb* liver schizonts IC_50_	46 nM	0.92 nM
	*Pv* liver hypnozoites/schizonts IC_50_	<100 nM	<100 nM
	*Pf*/*Pv ex vivo*	202 nM	29 nM
	*Pf* early gametocytes IC_50_	215 nM	134 nM
	*Pf* late/mature gametocytes IC_50_	140 nM	66 nM
	*Pf g*amete (male/female) IC_50_	91/139 nM	83/87 nM
	*Pf* oocyst reduction IC_50_	111 nM (indirect)	96 nM
efficacy	*Pb* ED_90_ (NOD-*scid IL-2R*_*γ*_^*null*^ mouse)	1.1 mg·kg^–1^	1.0 mg·kg^–1^
	*Pf* ED_90_ (NOD-*scid IL-2R*_*γ*_^*null*^ mouse)	0.57 mg·kg^–1^	0.25 mg·kg^–1^

Although MMV048 and UCT943 share the same MoA, UCT943 displayed better *in vitro* potency against both drug sensitive and resistant strains of *Pf* as well as higher transmission blocking and liver stage activities. We believe that UCT943’s attributes like superior solubility, high passive permeability translating to higher bioavailability, and sustained exposure are the main reasons behind its superior activity compared to MMV048.^21^Table 1 shows a comparative summary of various *in vitro* and *in vivo* properties of MMV048 and UCT943. Having had cleared toxicology and PK studies in different species, UCT943 was approved as a preclinical candidate in 2016 with promising potential to form part of a single-exposure radical cure and prophylaxis treatment of uncomplicated malaria.^19,21^

## Tuberculosis

With an established medium-throughput screening platform for both phenotypic and target-based screening, the H3D TB portfolio is made up of H2L and LO programs underpinned by a series of novel chemical matter. Historically, the whole-cell screening approach has been more successful in delivering active small molecules as starting points for TB drug discovery. We largely utilized this approach with hits being progressed through the various drug discovery stages, mainly supported by cell-based medicinal chemistry optimization, toward improving potency and pharmacokinetic/pharmacodynamic properties while also minimizing toxicity. We aim to identify a novel chemical class, targeting a novel molecular target. And to achieve this, as described above, in parallel to the medicinal chemistry and pharmacological studies, we perform hit-triaging at an early stage to avoid rediscovery of established targets and/or MoAs.^22^ Additionally, we have learned the importance of MoA studies in driving successful SAR exploration.

**Figure 4:**
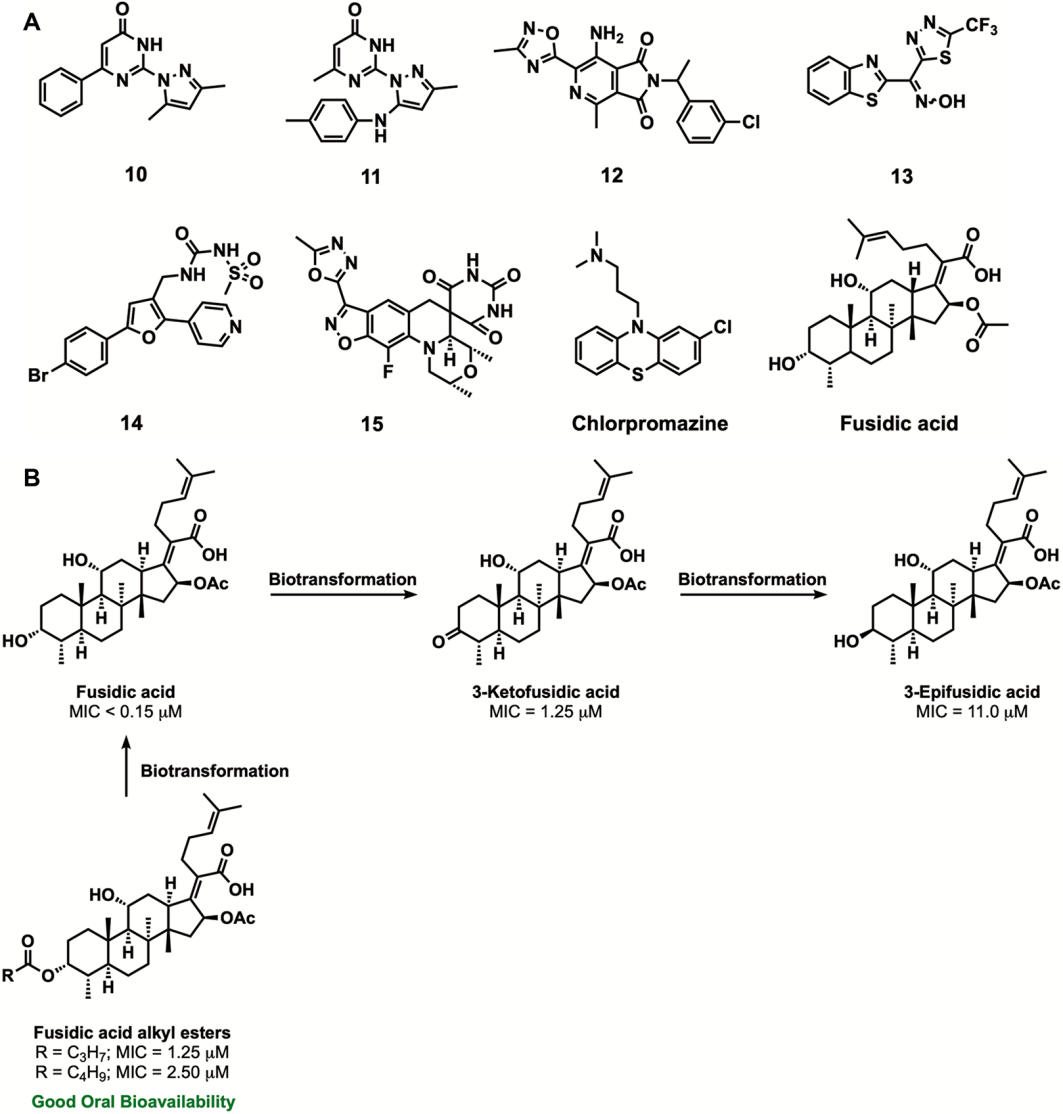
Chemical leads of the H3D tuberculosis portfolio. (A) Structures of compounds **10**–**15**, chlorpromazine, and fusidic acid. (B) An integrated approach of investigating fusidic acid for TB via SAR, biotransformation, and DMPK.^22,25−27^ Reproduced from ref (22). Copyright 2021 American Chemical Society.

In one such example, a high-throughput phenotypic screen of a MMV library comprising an ∼530,000 diverse set of compounds against *Mycobacterium tuberculosis* (*Mtb*) yielded active hits. This was followed by evaluating these hits in triaging assays that constitute additional critical assays which are performed during hit selection and are aimed at confirming the selectivity of the large number of active hits and at the same time help in understanding the MoA. As the choice of carbon source, Fe, albumin, and the detergent used were reported to have a profound effect on the efficacy of compounds,^23^ multiple growth media conditions were utilized to profile the minimum inhibitory concentrations (MICs) of the confirmed hits that are represented by a cluster of pyrazolylpyrimidinones (**10** and **11**; Figure 4).^24^

SAR studies resulted in compounds with improved potency against *Mtb*, excellent *in vitro* microsomal stability, and moderate to high aqueous solubility. Time-kill kinetics revealed the bactericidal nature of compounds against replicating *Mtb*, showing 2 log CFU reduction at 1–2 × MIC over the time period of 7 days. The pyrazolylpyrimidinones were effective against clinical isolates. Next, compounds were profiled against a mutant of the cytochrome *b* subunit of the cytochrome-*bc1* complex (QcrB^A396T^) and against a cytochrome-*bd* oxidase knockout mutant strain; no MIC modulation eliminated these as potential targets. Furthermore, the compounds did not yield a positive signal in two standard bioluminescence reporter assays of cell-wall damage and genotoxicity. A *Mtb* strain carrying a mutation in the promiscuous decaprenylphosphoryl-β-d-ribose 2′-epimerase (DprE1^C387S^) was not resistant to the compounds, suggesting DprE1 is not the target. However, selected strains carrying mutations in another promiscuous target mycobacterial membrane protein Large 3 (MmpL3^F255L^ or MmpL3^V681I^ or MmpL3^G596R^) showed cross-resistance to the compounds. Interestingly, there was no change in activity against the MmpL3^F644L^ mutant. To investigate whether pyrazolylpyrimidinones retain target selectivity for MmpL3 in *Mtb* cells, we asked whether conditional silencing of *mmpL3* would sensitize *Mtb* to the growth inhibitory effects of the pyrazolylpyrimidinones. To our surprise, there was no MIC modulation. To this end, as most of these mutations lie within the region required for proton translocation, we hypothesized that MmpL3 acts as a transporter of these compounds across the cell membrane as the compounds can form heme-like iron-complexes, and MmpL3 is known to act as a heme transporter. Next, we performed transcription analyses of *Mtb* cultures treated with pyrazolylpyrimidinones. This revealed the upregulation of genes involved in iron-homeostasis, further confirming the finding of Poirier et al. that pyrazolylpyrimidones act by via metal chelation.^28^ This was further verified in a 2D-checkerboard assay by iron supplementation to the growth medium displaying rescue of bacterial growth from the toxicity of pyrazolylpyrimidinones, confirming the perturbation of Fe-homeostasis as a MoA. This highlights the need to include such metal chelating groups among pan-assay interference compounds.^29^ Due to the challenges in improving the selectivity index between MIC and mammalian cytotoxicity, further work on the series has been discontinued.

**Figure 5:**
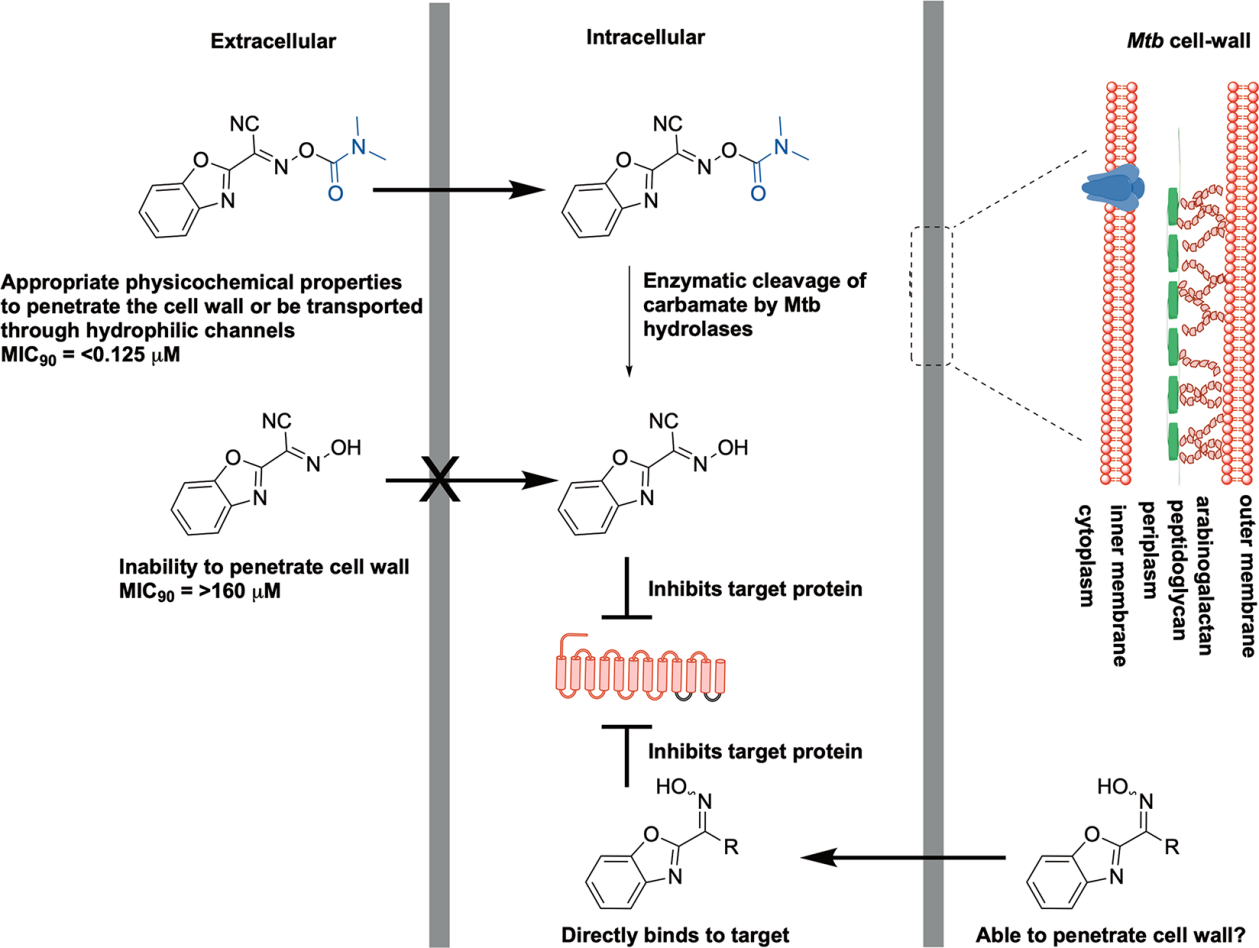
Proposed prodrug-based activity of carbamate-functionalized oxime compounds.^31^ Reproduced from ref (31). Copyright 2021 American Chemical Society.

In another whole-cell screening campaign, two potent hit series (MIC of <0.5 μM), the pyrrolo[3,4-*c*]pyridine-1,3(2*H*)-diones exemplified by compound **12**(30) and benzoheterocyclic oxime carbamates represented by compound **13**,^31^ were identified (Figure 4). The oxime carbamate containing compounds displayed potent activity (MIC < 0.08–0.31 μM) against drug-susceptible clinical *Mtb* isolates. The hits displayed strong selectivity toward mycobacteria but were inactive (MIC > 125 μM) against a panel of five Gram negative and one Gram positive bacterial pathogens. Encouragingly, this series exhibited good selectivity when tested on mammalian Chinese hamster ovary cells at concentrations of 50 μM. During SAR, cytotoxicity, solubility, and ADME/PK profiling, it was discovered that while the parent carbamates maintained activity, the free oximes were inactive (Figure 5).^31^ To investigate this, we hypothesized that the carbamate group masks the oxime in the compounds to improve permeation across the *Mtb* cell wall. Once the compounds are in the bacilli, these can easily be enzymatically cleaved via esterase activity. Indeed, experiments involving compound incubation with *Mtb* cell-lysate confirmed that this series acts as a prodrug for *Mtb*.

In another high-throughput phenotypic screening campaign, an ∼150,000-member agrochemical library of a diverse set of compounds from DuPont was screened against *Mtb* in cholesterol-containing media. One of the moderately active hit, 1,3-diarylpyrazolyl-acylsulfonamide (MIC ∼ 5 μM), was explored by SAR to improve whole-cell potency to MIC values of ∼0.15 μM (compound **14**, Figure 4).^32^ Compounds were bactericidal against replicating *Mtb* and retained potency against drug-resistant *Mtb* clinical isolates. Biology triage assays suggested the involvement of cell-wall biosynthesis in the MoA. However, cross-resistance profiling against the mutants of the known cell-wall targets such as MmpL3, DprE1, InhA (target of isoniazid, encoding enoyl-[acyl-carrier-protein] reductase), and EthA (monooxygenase, activating ethionamide) was suggestive of the novel MoA. Our current efforts are focused on validating the MoA and establishing the *in vivo* efficacy of this promising series.

DNA gyrase in *Mtb* is a validated target of fluoroquinolones; inhibition of DNA gyrase after DNA cleavage results in permanent double-strand DNA breaks and impaired replication. Interestingly, the *Mtb* DNA gyrase inhibitor moxifloxacin failed to shorten the treatment duration in a Phase III trial;^33^ this could be due to the inadequate spatial distribution of moxifloxacin in intact lesions to kill nonreplicating *Mtb*.^34^ Nonetheless, in a recent study, the combination of moxifloxacin with rifapentine has shown potential for treatment shortening and validating the clinical relevance of DNA gyrase.^35^ To this end, we selected spiropyrimidinetriones, a new class of antibacterial agent that inhibits DNA gyrase in a unique way compared to fluoroquinolones in other bacteria.^36^ We hypothesized that spiropyrimidinetriones would inhibit *Mtb* DNA gyrase in a similar way to moxifloxacin and that whole-cell activity against *Mtb* would be cidal. Spiropyrimidinetrione analogues, obtained from Entasis Therapeutics, were accordingly screened against *Mtb* under various culture conditions.^37^ Compound **15** displayed a range of MICs (1.7–5.2 μM; with the minimum bactericidal concentration being only 2-fold higher than its MIC) in different growth media, and the lack of cross-resistance to various antitubercular drug-resistant *Mtb* mutants underpins the importance of spiropyrimidinetriones for eventual stewardship to the clinic. Importantly, *Mtb* strains resistant to fluoroquinolones were fully susceptible to spiropyrimidinetriones; this is attributed to the spiropyrimidinetrione class operating via a novel mode of inhibition, which involves Mg^2+^-independent stabilization of the DNA cleavage-complex with DNA gyrase. However, compound **15** exhibited a weaker MIC compared to moxifloxacin despite showing better DNA gyrase inhibition activity than moxifloxacin.^37^ This guided us toward design efforts to optimize spiropyrimidinetrione bacterial permeability and target potency.

To tackle drug resistance and potentially reduce the cost/time of drug development, our efforts also involved drug repositioning or repurposing of clinically approved drugs.^38^ In this context, we investigated chlorpromazine (Figure 4), a phenothiazine for treatment of psychosis, and observed its synergy with spectinomycin, kanamycin, streptomycin, and with an active metabolite of rifampicin (25-desacetylrifampicin).^39^ We also explored fusidic acid (FA, Figure 4) which displayed good activity against both drug susceptible and resistant clinical *Mtb* isolates,^40^ qualifying as a viable candidate for repositioning. We worked on the SAR,^25,26^ studied biotransformation,^26^ and by using a prodrug approach improved the absorption and tissue distribution of FA (Figure 4B).^27^ Next, by using chemical biology and genetics, we identified and confirmed the molecular target of FA in *Mtb* as elongation factor G encoded by *fusA1.* To validate FusA1 as a novel drug target in *Mtb*, we also tested the viability of *fusA1* conditional knockdown upon *fusA1* silencing. This resulted in the cidality of *Mtb* both *in vitro* and in macrophages, confirming FusA1 as a novel, chemically tractable, and vulnerable target in *Mtb*.^41^ Owing to the attractiveness of drug repositioning and repurposing approaches, our efforts are continuing in this direction.

The innovation journey of H3D is one of many other notable examples of the changing paradigm for research on the African continent. While there are several challenges that still need to be addressed, progress is clearly being made. MMV048 discovered by an international team led by H3D is the first candidate that has been used not only as a tool compound for target identification but also possesses drug-like characteristics. When MMV048 entered human clinical trials (Phase Ia in 2014, Phase Ib in 2016 and Phase IIa in 2017), it became the first antimalarial developed on the African soil to reach human clinical trials. Discovered from phenotypic high-throughput screening and progressed through cell-based medicinal chemistry optimization, MMV048 was earlier shown to be an exceptional candidate endowed with single-dose curative effects in mouse infection models of malaria. Its effect spans across a panel of resistance strains with a novel MoA. Despite the further clinical development of MMV048 being stopped in Phase II due to some preclinical safety liabilities, which are yet to be understood, the discovery process of MMV048 and UCT943 facilitated not only the advancement of basic and clinical sciences but also infrastructure development which to this day partly anchors the drug discovery capabilities at H3D.^13^ MoA studies leading to the identification of *Pf*PI4K as the novel target of MMV048 ushered the first time in which a chemical proteomics approach was used to identify a malaria drug target. At this juncture, it is noteworthy that MMV048 has set the precedence for the clinical validation of a *Plasmodium* kinase inhibitor.^13^

With TB, our experiences informed us of the use of diverse chemical libraries for screening, the importance of media compositions, the use of innovative screening approaches, and appropriate animal models. In addition, for TB, we have learned to frontload compound metabolic stability studies in the presence of *Mtb* to address *Mtb*-mediated drug metabolism early on during the drug discovery process. While we have yet to deliver a preclinical/clinical candidate for TB, we are encouraged with the progress on an ongoing project that has moved beyond the lead optimization phase. Finally, we call on further contributions from across the continent to this effort, to grow our own timber, reverse the brain-drain, and equip our continent to be an equal contributor to drug discovery and the advancement of translational medicine across the globe.

## Abbreviations

PDPs: product development partnerships
MMV: Medicines for Malaria Venture
MalDA: Malaria Drug Accelerator
TBDA: TB Drug Accelerator
*Pf*: *Plasmodium falciparum*
NF54: drug sensitive *Pf* strain
K1: multidrug resistant *Pf* strain
3D7: drug sensitive *Pf* strain
Dd2: drug-resistant *Pf* strain
SFK: SoftFocus Kinase library
PI4K: phosphatidylinositol-4-kinase
TB: tuberculosis
*Mtb*: *Mycobacterium tuberculosis*
